# *SOCS3* genetic variants and promoter hypermethylation in patients with chronic hepatitis B

**DOI:** 10.18632/oncotarget.15083

**Published:** 2017-02-04

**Authors:** Nghiem Xuan Hoan, Hoang Van Tong, Dao Phuong Giang, Bui Khac Cuong, Nguyen Linh Toan, Heiner Wedemeyer, C. Thomas Bock, Peter G Kremsner, Le Huu Song, Thirumalaisamy P Velavan

**Affiliations:** ^1^ Institute of Tropical Medicine, University of Tübingen, Tübingen, Germany; ^2^ Institute of Clinical Infectious Diseases, 108 Military Central Hospital, Hanoi, Vietnam; ^3^ Vietnamese-German Center for Medical Research (VG-CARE), Hanoi, Vietnam; ^4^ Department of Pathophysiology, Vietnam Military Medical University, Hanoi, Vietnam; ^5^ German Center for Infection Research, Department for Gastroenterology, Hepatology, and Endocrinology, Medical School Hannover, Germany; ^6^ Department of Infectious Diseases, Robert Koch Institute, Berlin, Germany; ^7^ Faculty of Medicine, Duy Tan University, Da Nang, Vietnam

**Keywords:** HBV infection, liver diseases, SOCS3 variants, SOCS3 methylation

## Abstract

The clinical manifestations of hepatitis B viral infection (HBV) include chronic hepatitis B (CHB), liver cirrhosis (LC) and hepatocellular carcinoma (HCC). The contribution of negative regulator suppressor of cytokine signaling-3 (SOCS3) promoter variants in HBV disease and *SOCS3* hypermethylation in tumor tissues were investigated The *SOCS3* promoter region was screened for polymorphisms in 878 HBV patients and in 272 healthy individuals. *SOCS3* promoter methylation was examined by bisulfite sequencing. *SOCS3* mRNA expression was quantified in 37 tumor and adjacent non-tumor liver tissue specimens. The minor allele *rs12953258A* was associated with increased susceptibility to HBV infection (OR=1.3, 95%CI=1.1-1.6, adjusted *P*=0.03). The minor allele *rs111033850C* and *rs12953258A* were observed in increased frequencies in HCC and LC patients compared to CHB patients (HCC: OR=1.7, 95%CI=1.1-2.9, adjusted *P*=0.046; LC: OR=1.4, 95%CI=1.1-1.9, adjusted *P*=0.017, respectively). HBV patients with *rs111033850CC* major genotype had decreased viral load (*P*=0.034), whereas the *rs12953258AA* major genotype contributed towards increased viral load (*P*=0.029). Tumor tissues revealed increased hypermethylation compared to adjacent non-tumor tissues (OR=5.4; 95%CI= 1.9-17.1; *P*=0.001). Increased *SOCS3* expression was observed in HBV infested tumor tissues than non-HBV related tumor tissues (*P*=0.0048). *SOCS3* promoter hypermethylation was associated with relatively low mRNA expression in tumor tissues (*P*=0.0023). In conclusion, *SOCS3* promoter variants are associated with HBV susceptibility and SOCS3 hypermethylation stimulates HCC development.

## INTRODUCTION

Hepatitis B virus (HBV) infection is a major health problem affecting approximately two billion people worldwide. Approximately 240 million individuals are chronically infected with 780,000 annually reported deaths due to HBV infection [1]. HBV infection causes a wide spectrum of clinical manifestations of liver diseases. Besides asymptomatic carriers, HBV causes chronic hepatitis B (CHB), liver cirrhosis (LC) and hepatocellular carcinoma (HCC). The five-year cumulative risk for the development of HBV-related LC ranges between 10% and 20% [2]. In addition, chronic HBV infection accounts for 50% of all HCC cases and most HCC cases (70%-80%) occur in patients with HBV-related LC [3].

During the course of HBV infection, the mechanism of liver injury is dependent on the host immune responses [4]. The innate immune responses play a major role in suppression of viral replication and in inflammatory activity during the early stage of HBV infection. These responses include the secretion of interferons (IFNs) and cytokines, which are regulated by Janus kinase/Signal Transducer and Activator of Transcription (JAK/STAT) signaling [5, 6] and involvement of JAK/STAT pathway in HBV infection had been well documented [7, 8]. Suppressors of cytokine signaling (SOCS) family proteins (CISH and SOCS1 to SOCS7) belong to a classical negative feedback system that regulates cytokine transduction via JAK/STAT signaling pathway [9]. Of these SOCS family, SOCS3 is a key regulator of interleukin (IL)-6 and IL-10, which are activated by Toll-like receptor stimulation. SOCS3 can inhibit the process of cell proliferation and cell survival through inhibition of STAT3 activation [6, 10]. *STAT3*, an oncogene, is largely correlated with NF-kβ activation [11, 12]. The activation of STAT3 by cytokines (e.g. IL-6 and IL-22) mediated by JAK/STAT signaling pathway was shown earlier to induce inflammation and subsequent carcinogenesis [10–12]. SOCS3 functionally suppresses STAT3 activation and negatively regulate tumor development. Therefore, SOCS3 is a vital regulator of several diseases including atopic, autoimmune and infectious diseases, inflammation, and cancer development [13, 14].

SOCS3 overexpression has been shown in the liver tissue of CHB patients and is associated with the severity of inflammation suggesting that JAK/STAT signaling pathway is dysregulated in HBV-infected hepatocytes [15]. The hypermethylation in the CpG (5′-Cytosine-phosphate-Guanine-3′) islands of the *SOCS3* promoter can be a prognostic indicator in cancer development [16]. In addition, *SOCS3* expression is further influenced by *SOCS3* polymorphisms, especially in the *SOCS3* promoter. To date, *SOCS3* polymorphisms were documented in several diseases including HCV infection and colorectal cancer [14, 17–20]. However, there are so far no available data on association of *SOCS3* promoter variants with susceptibility to HBV infection and the clinical course of HBV-related liver diseases. In addition, the involvement of epigenetics during the clinical course of HBV infection needs to be studied. Therefore, this study aims to investigate whether *SOCS3* promoter variants are associated with HBV infection and HBV-related liver diseases and to investigate the hypermethylation in the *SOCS3* promoter region and corresponding *SOCS3* mRNA expression in HBV-related HCC.

## RESULTS

### Baseline characteristics of study participants

The baseline characteristics of the 878 HBV-infected patients and 272 healthy controls (HC) are shown in Table 1. Most HBV patients and HCs were male (86% and 66%, respectively). The median age of patients increased according to the clinical progression of the liver disease. HCs were younger than patient groups (*P*<0.05). The levels of liver enzymes ALT, AST and HBV loads were higher in CHB patients compared to other subgroups (*P*<0.01). As expected, albumin and prothrombin levels and platelet counts were lower in LC patients compared to the other patient groups (*P*<0.001). AFP levels were higher in HCC patients compared to CHB and LC patients (*P*<0.001). The clinical profile of the 37 HCC patients who underwent surgery and corresponding data of their liver specimens are described in Table 2. Most patients were male (89%) and were between 40-60 years of age (73%). The HCC patients were in early and/or at intermediate stage of liver cancer according to the Barcelona clinic liver cancer (BCLC) staging criteria (stage A: 70% and stage B: 30%). All the patients were Child-Pugh class A group according to Child-Pugh classification. HBV was the common etiology of the liver cancer in this study (46%), while 8% suffered from HCV infection, and 46% showed non-HBV/non-HCV related HCC.

**Table 1:** Clinical profiles of 878 HBV-infected patients and 272 healthy controls

Characteristics	CHB (n=212)	LC (n=243)	HCC (n=220)	LC + HCC (n=203)	HC (n= 272)
Age (years)	43 [18-82]	55 [18-84]	55 [18-81]	50 [19-81]	36 [18-69] ^‡α^
Male (%)	74	84.4	92.7	94.1	66 ^†α^
Child-Pugh classification (n)					
Child A	NA	117/236	101/173	93/164	NA
Child B	NA	81/236	56/173	52/164	NA
Child C	NA	38/236	16/173	19/164	NA
Missing	NA	7	47	39	NA
Clinical parameters					
AST (IU/L)	72 [15-3253] ^‡β^	52 [15-1221]	50 [17-2158]	49 [21-737]	NR
ALT (IU/L)	69 [9-3382] ^‡β^	46 [8-1426]	46 [10-832]	44 [10-1095]	NR
Total bilirubin (μmol/l)	17 [8-788]	29 [3-752]	14 [5-235]	22 [7-419]	NR
Direct bilirubin (μmol/l)	7 [1-472]	12 [1-450]	5 [1.2-167]	8 [1-214]	NR
Albumin (g/L)	42 [23-48]	30 [20-47]^‡β^	39 [27-49]	38 [23-47]	NR
Prothrombin (% of standard)	87 [30-180]	53.5 [15-101]^§β^	80 [31-115]	74 [19.6-118]	NR
PLT (10^3^/ml)	208 [19-360]	90 [18-441]^§β^	203 [20-389]	122 [34-361]	NR
HBV-DNA (copies/ml)	1.6x10^7^ [2x10^2^- 8.4x10^10^]^§β^	6.8x10^4^ [1.8x10^2^- 4.7x10^9^]	7.4x10^5^ [2.9x10^2^-1.4x10^9^]	1.6x10^5^ [1.9x10^2^- 3.1x10^10^]	NA
Alfa Feto Protein (IU/L)	4.3 [1.5-300]	7.4 [1.2-400]	196 [1.1- 438] ^§β^	168 [1.6-489] ^§β^	NR

Abbreviations: CHB: chronic hepatitis B; LC: liver cirrhosis; HCC: hepatocellular carcinoma; HC: healthy control; PLT: platelets. AST and ALT: aspartate and alanine amino transferase; IU: international unit; NR: Normal range, NA: not applicable. Values given are medians and range. *P* values were calculated by student's t-test, Fisher exact test, and Mann-Whitney- Wilcoxon test where appropriate. (†) *P*<0.05, (‡) *P*<0.01 and (§) *P*<0.001. (α) for comparison with LC, HCC and LC+HCC group; (β) for comparison with all other groups.

**Table 2:** Characteristics of 37 HCC patients

Characteristics	n (%)
**Age (years)**	
< 40	4/37 (10.8)
40 - 60	27/37 (73)
> 60	6/37 (16.2)
**Gender**	
Male	33/37 (89.2)
Female	4/37 (10.8)
**Etiology**	
HBV	17/37 (46)
HCV	3/37 (8)
Non-HBV/HCV	17/37 (46)
**Child-Pugh classification**	
Child A	37/37 (100)
**BCLC staging Classification**	
Stage A	26/37 (70.3)
Stage B	11/37 (29.7)
Stage C and D	0/ 37 (0)
**Clinical parameters**	**Median (Range)**
AFP (IU/ml)	240 [4.6 - 300]
HBV-DNA	NA
PLT (10^3^/ml)	211 [153 - 461]
AST (IU/ml)	52 [21 - 415]
ALT (IU/ml)	66.5 [17 - 242]
Total Bilirubin (μmol/l)	27.8 [8.9 - 315]
Direct Bilirubin (μmol/l)	6.7 [1 - 178]
Prothrombin (% of standard)	93 [75 - 125]
Protein (g/l)	73 [62 - 78]
Allbumin (g/l)	40 [32 - 48]

Abbreviations: BCLC: Barcelona Clinic Liver Cancer; HCC: hepatocellular carcinoma; AFP: Alpha feto protein; PLT: platelets; AST and ALT: aspartate and alanine amino transferase; IU: international unit; NA: not applicable.

### *SOCS3* promoter variants and HBV-related liver diseases

The genotype and allele frequencies of two *SOCS3* promoter SNPs (rs111033850T/C, rs12953258C/A) in clinically classified 878 HBV patients and 272 HCs are described in Table 3 and Supplementary Table 2. The analyzed SNPs in healthy controls were in Hardy-Weinberg equilibrium (*P*>0.05). We compared the genotype and allele frequencies between HBV patients and HCs. We observed that heterozygous genotype *rs111033850TC* and minor allele *rs111033850C* were less frequent in HBV patients compared to HCs (OR=0.4, 95%CI=0.3-0.6, adjusted *P*<0.0001 and OR=0.6, 95%CI=0.4-0.8, adjusted *P*<0.0001; respectively). In contrast, the homozygous genotype *rs12953258AA* and the minor allele *rs12953258A* were more frequent in patients compared to HCs (OR=2.0, 95%CI=1.3-3.2, adjusted *P*<0.0001 and OR=1.3, 95%CI=1.1-1.6, adjusted *P*=0.03; respectively). A similar trend was observed for the SNP rs111033850T/C in the dominant genetic model and for the SNP rs12953258C/A in the recessive genetic model. These results indicated that the *rs111033850TC* contributes to a decreased risk of HBV infection while the genotype *rs12953258AA* contributes to increased susceptibility to HBV infection.

**Table 3:** Association of *SOCS3* variants with HBV-related liver diseases

*SOCS3*variants	CHBn (%)	LCn (%)	HCCn (%)	HCC+LCn (%)	HCn (%)	Cases vs. HC		LC vs. CHB		HCC vs. CHB		HCC+LC vs. CHB	
	n=212	n=243	n=220	n=203	n=272	OR(95%CI)	*P* value	OR(95%CI)	*P* value	OR(95%CI)	*P* value	OR(95%CI)	*P* value
**rs111033850**													
*TT*	190 (89.6)	198 (81.5)	178 (80.9)	162 (79.8)	191 (70.3)	Reference		Reference		Reference		Reference	
*TC*	16 (7.5)	43 (17.7)	36 (16.4)	35 (17.2)	76 (27.9)	**0.4 (0.3-0.6)**	**<0.0001**	**2.6 (1.4-5.0)**	**0.002**	**2.6 (1.3-5.0)**	**0.005**	**2.0 (1- 4.4)**	**0.048**
*CC*	6 (2.9)	2 (0.8)	6 (2.7)	6 (3.0)	5 (1.8)	1.1 (0.4 -3.0)	0.89	0.4 (0.1-2.0)	0.2	0.9 (0.2-3.1)	0.8	1.2 (0.3 - 5.3)	0.8
*P* for trend							1.99		0.12		**0.046**		**0.026**
**Allele**													
*T*	396 (93.4)	439 (90.3)	392 (89)	359 (88.4)	458 (84.2)	Reference		Reference		Reference		Reference	
*C*	28 (6.6)	47 (9.7)	48 (11)	47 (11.6)	86 (15.8)	**0.6 (0.4-0.8)**	**<0.0001**	1.6 (0.9-2.6)	0.07	**1.7 (1.1-2.9)**	**0.046**	1.7 (0.9-3.0)	0.09
**Dominant**													
*TT*	190 (89.6)	198 (81.5)	178 (80.9)	162 (79.8)	191 (70.3)	Reference		Reference		Reference		Reference	
*TC & CC*	22 (10.4)	45 (18.5)	42 (19.1)	41 (20.2)	81 (29.7)	**0.5 (0.3-0.7)**	**<0.0001**	**2.0 (1.1-3.7)**	**0.014**	**2.0 (1.1-3.8)**	**0.017**	**1.9 (1.1-3.7)**	**0.046**
**Recessive**													
*TT & TC*	206 (97.2)	241 (99.2)	214 (97.3)	197 (79.8)	267 (98.2)	Reference		Reference		Reference		Reference	
*CC*	6 (2.9)	2 (0.8)	6 (2.7)	6 (3.0)	5 (1.8)	1.3 (0.5-3.7)	0.64	0.3 (0.1-1.8)	0.2	0.8 (0.2-2.8)	0.7	1.1 (0.3-5.3)	0.8
*P* for trend							0.058		0.059		0.55		0.33
**rs12953258**													
*CC*	94 (44.3)	88 (36.2)	86 (39.1)	75 (36.9)	101 (37.1)	Reference		Reference		Reference		Reference	
*AC*	72 (34)	88 (36.2)	88 (40)	84 (41.4)	140 (51.5)	**0.7 (0.5-0.98)**	**<0.0001**	1.3 (0.8-2.0)	0.27	1.34 (0.8-2.2)	0.21	1.4 (0.8-2.4)	0.22
*AA*	46 (21.7)	67 (27.6)	46 (20.9)	44 (21.7)	31 (11.4)	**2.0 (1.3-3.2)**	**<0.0001**	**1.7 (1.02-2.8)**	**0.036**	1.0 (0.6-1.7)	0.96	1.4 (0.7-2.7)	0.26
**Allele**													
*C*	260 (61.3)	264 (54.3)	260 (59.1)	172 (42.4)	342 (62.9)	Reference		Reference		Reference		Reference	
*A*	164 (38.7)	222 (45.7)	180 (40.9)	234 (57.6)	202 (37.1)	**1.3 (1.1-1.6)**	**0.03**	**1.4 (1.1-1.9)**	**0.017**	0.9 (0.7 - 1.2)	0.87	1.3 (0.9-1.8)	0.15
**Dominant**													
*CC*	94 (44.3)	88 (36.2)	86 (39.1)	75 (36.9)	101 (37.1)	Reference		Reference		Reference		Reference	
*AC & AA*	118 (55.7)	155 (63.8)	134 (60.9)	128 (63.1)	171 (62.9)	1.0 (0.7-1.3)	0.72	1.45 (0.9-2.2)	0.07	1.2 (0.8-1.8)	0.38	1.4 (0.8-2.3)	0.16
**Recessive**													
*CC & AC*	166 (78.3)	176 (72.4)	174 (79.1)	159 (78.3)	241 (88.6)	Reference		Reference		Reference		Reference	
*AA*	46 (21.7)	67 (27.6)	46 (20.9)	44 (21.7)	31 (11.4)	**2.4 (1.6-3.7)**	**<0.0001**	1.5 (0.9-2.4)	0.08	0.9 (0.5-1.5)	0.61	1.2 (0.7-2.1)	0.47

Abbreviations: CHB: Chronic hepatitis B; LC: Liver cirrhosis; HCC: Hepatocellular carcinoma; HC: Healthy control; Cases = all HBV infected patients; n= Number of chromosomes; OR: adjusted Odd Ratio; ORs and *P* values were calculated by using binary logistic regression model adjusted for age and gender. *P* for trend was calculated by Cochran-Armitage test. Bold values present the statistical significance.

Subsequently, we compared the genotype and allele frequencies between different subgroups of HBV patients. The genotype *rs12953258AA* was significantly more frequent in LC patients compared to CHB patients (OR=1.7, 95%CI=1.02-2.8; adjusted *P*=0.036). The genotype *rs111033850TC* was also significantly more frequent in LC, HCC and HCC+LC groups compared to CHB patients (LC vs. CHB: OR=2.6, 95%CI=1.4-5.0, adjusted *P*=0.002; HCC vs. CHB: OR=2.6, 95%CI=1.3-5.0, adjusted *P*=0.005; LC+HCC vs. CHB: OR=2.0, 95%CI=1-4.4, adjusted *P*=0.048). The alleles *rs111033850C* and *rs12953258A* were more frequent in HCC and in LC patients compared to CHB patients, respectively (OR=1.7, 95%CI=1.1-2.9, adjusted *P*=0.046 and OR=1.4, 95%CI=1.1-1.9, adjusted *P*=0.017).

We also observed the gene dose effect of the allele *rs111033850C* when compared CHB with HCC and HCC+LC groups (*P* for trend =0.046 and 0.026, respectively). This indicate that the allele *rs111033850C* was associated with an increased risk of HCC and that the allele *rs12953258A* was associated an increased risk of LC in CHB patients. There were no significant differences when comparing the genotype and allele frequencies of the two *SOCS3* SNPs in LC and HCC groups with HCC+LC group.

### *SOCS3* promoter haplotypes and HBV-related liver diseases

The haplotypes were reconstructed based on the two SNPs (rs111033850T/C, rs12953258C/A) and the frequencies were presented in Table 4. Haplotype *CC* was found more frequently in HCs compared to HBV patients (OR=0.5, 95%CI=0.4-0.75, adjusted *P*=0.001) indicating that this haplotype contributes to a decreased risk of HBV infection. We further compared haplotype frequencies between different subgroups of HBV patients and observed that the frequencies of haplotypes *TA* and *CC* were significantly higher in LC, HCC and patients with both LC and HCC compared to CHB patients (*P*<0.01) (Table 4). This result indicates that the haplotypes *TA* and *CC* contribute to an increased risk of progression to LC and HCC in HBV patients. However, no significant difference was observed when haplotype frequencies of LC and HCC patients were compared.

**Table 4:** Association of *SOCS3* haplotypes with HBV-related liver diseases

*SOCS3*Haplotype	HC	CHB	LC	HCC	HCC+LC	Cases vs. HC		LC vs. CHB		HCC vs. CHB		HCC+LC vs. CHB	
	n=544	n= 424	n= 486	n= 440	n= 406	OR (95%CI)	*P*	OR (95%CI)	*P*	OR (95%CI)	*P*	OR (95%CI)	*P*
*TC*	*256 (47.0)*	*244 (57.5)*	*216 (44.4)*	*204 (46.4)*	196 (48.3)	Reference		Reference		Reference		Reference	
*TA*	*194 (35.7)*	*154 (36.3)*	*228 (46.9)*	*186 (42.3)*	162 (39.9)	1.2 (0.9-1.5)	0.17	***1.89 (1.4-2.54)***	**0.0001**	***1.46 (1.1 - 2.0)***	**0.017**	**1.49 (1.1-2.13)**	**0.026**
*CC*	92 (16.9)	24 (5.7)	*42 (8.7)*	46 (10.5)	46 (11.3)	***0.5 (0.4 - 0.8)***	**0.001**	***2.1 (1.2-3.9)***	**0.008**	***2.76 (1.5 -5)***	**0.001**	***2.49 (1.3-4.8)***	**0.007**
*CA*	2 (0.4)	2 (0.5)	0 (0)	4 (0.9)	2 (0.5)	*0.9 (0.2 - 4.8)*	0.96	NA	NA	*1.89 (0.4-11.6)*	0.49	*1.47 (0.1 - 17)*	0.75

Abbreviations: CHB: Chronic hepatitis B; LC: Liver cirrhosis; HCC: Hepatocellular carcinoma; HC: Healthy control; Cases = all HBV infected patients; n= Number of chromosomes; NA: not applicable; OR: Adjusted Odd Ratio; ORs and *P* values were calculated by using binary logistic regression model adjusted for age and gender. Bold values present the statistical significance.

### *SOCS3* promoter variants and clinical parameters

**Figure 1:**
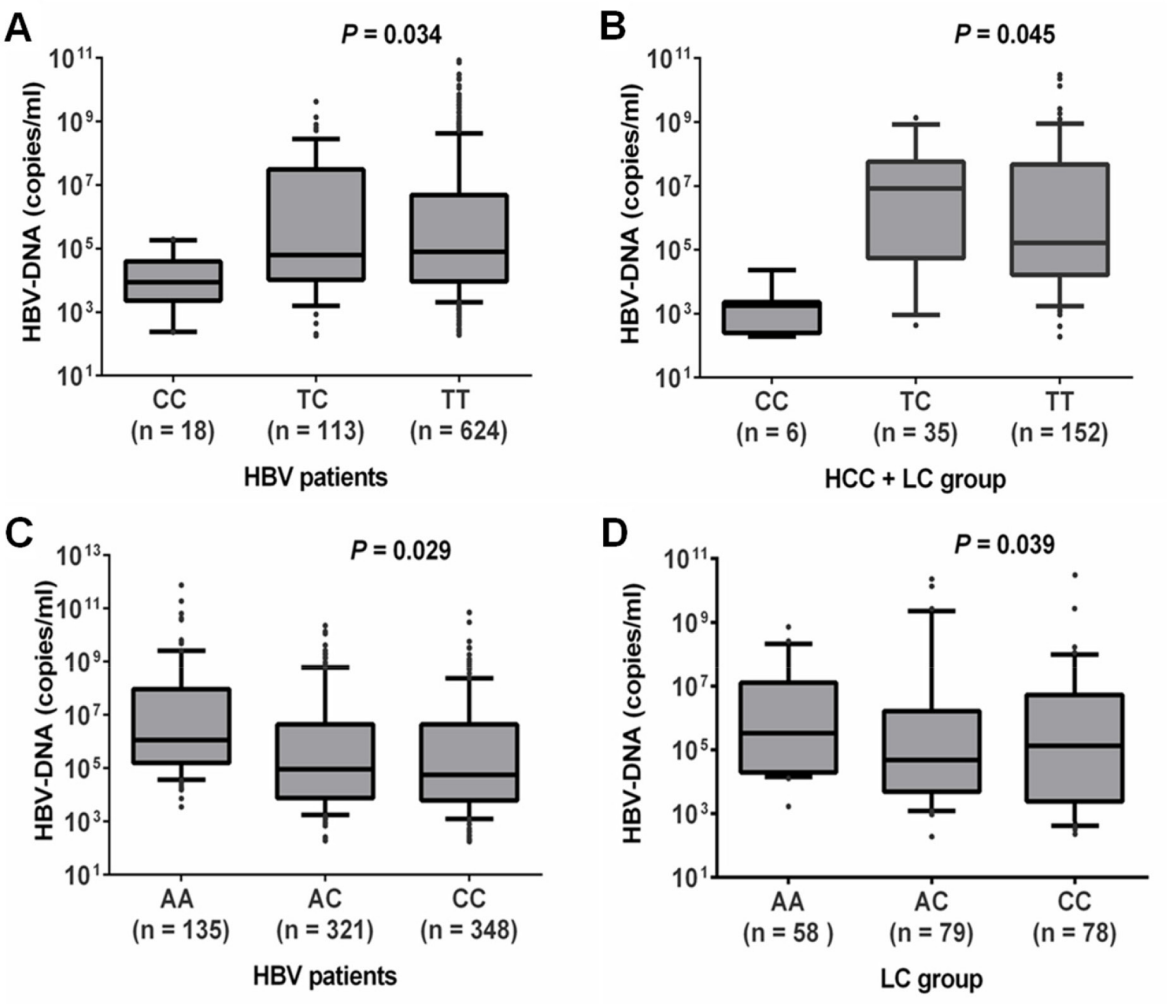
Association of HBV loads with SOCS3 SNPs **A**. and **B**. HBV viral loads according to different genotypes of SNP rs111033850T/C in all HBV patients and in patients with both liver cirrhosis and hepatocellular carcinoma, respectively. **C**. and **D**. HBV loads according to different genotypes of SNP rs12953258C/A in all HBV patients and in patients with liver cirrhosis, respectively. Box-plots illustrate medians with 25 and 75 percentiles with whiskers to 10 and 90 percentiles; *P* values were calculated by Krusskal-Wallis test.

The HBV loads were lower in HBV patients with *rs111033850CC* compared to those with *rs111033850TT* and *rs111033850TC* (*P*=0.034). In contrast, viral loads were higher in HBV patients with *rs12953258AA* compared to those with *rs12953258CC* and *rs12953258AC* (*P*=0.029) (Figure 1A and 1C). To further examine this possible association, we compared HBV loads according to different genotypes for each SNP in the subgroups of HBV patients. A similar trend of HBV loads was observed in HCC+LC group for rs111033850 (*P*=0.045) and in LC group for rs12953258 (*P*=0.039) (Figure 1B and 1D). However, there was no significant association of these SNPs with clinical parameters: ALT, AST, total and direct bilirubin, albumin, prothrombin, platelet counts and AFP (*P*>0.05) (Supplementary Figure 1 and 2). In addition, we also compared clinical parameters according to different haplotypes however we did not observe any significant association of *SOCS3* promoter haplotypes with laboratory parameters.

### Methylation status of *SOCS3* promoter region in primary HCCs

**Figure 2:**
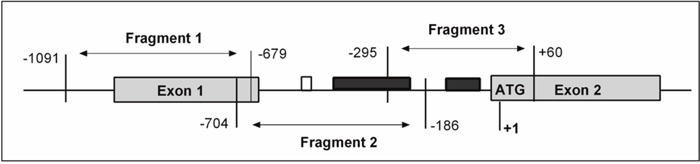
Schematic structure of *SOCS3* gene presenting the location of CpG island Methylation status of *SOCS3* promoter region was analyzed by bisulfite sequencing (BS). The start codon site for *SOCS3* gene is defined as +1. The shaded boxes depict the exons of the *SOCS3* gene and open box represents the reported STAT3-binding site and the black boxes represent the region with aberrant methylation in the fragments 2 and 3.

**Figure 3:**
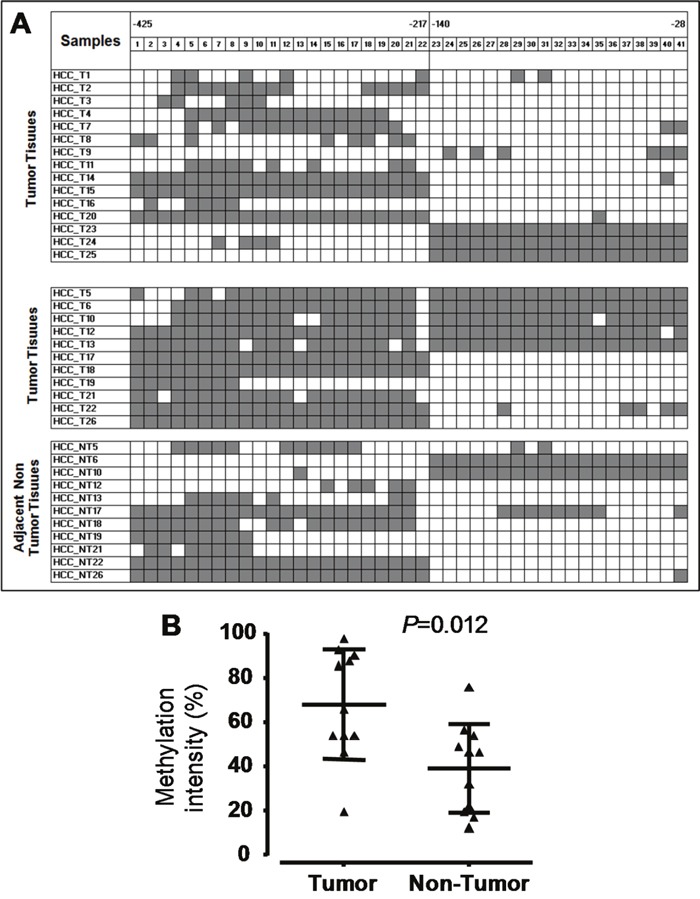
Methylation status of the *SOCS3* CpG islands in the promoter region **A**. Methylation status of HCC tumor and non-tumor tissues. We randomly sequenced five to eight clones of PCR products amplified from Bisulfite treated genomic DNA for each liver tissue sample. The highly methylated CpG islands were found in two regions (from positions -425 to -217 and from positions -140 to -28). Each square shape presents one CpG island, black color shows CpG islands with methylation and white color shows CpG islands without methylation. **B**. Methylation intensity of the 11 HCC tumor and 11 adjacent non-tumor tissue samples. *P* values were calculated by Mann-Whitney test.

In total, 127 CpG islands in the fragment of 1150 bp (-1091 through +60) in the *SOCS3* promoter region were investigated. We found that CpG islands were unmethylated in the fragment 1 (-1091 through -679) while were hypermethylated in the fragment 2 (-425 through -217) and in the fragment 3 (-140 through -28) (Figure 2 and 3A). Subsequently, we analyzed methylation status in the *SOCS3* promoter in 37 pairs of tissue samples (tumor and adjacent non-tumor tissues). We observed that the *SOCS3* promoter region was methylated in 26/37 (70.3%) liver tumor tissues while only in 11/37 (29.7%) adjacent non-tumor tissues showed methylation patterns (OR=5.4, 95%CI=1.9-17.1, *P*=0.0011) (Figure 3A). This result indicates that *SOCS3* promoter methylation occurs more frequently in tumor tissues compared to adjacent non-tumor tissues. In addition, we analyzed the intensity of *SOCS3* promoter methylation in 11 pairs of tissue samples, in which *SOCS3* promoter methylation was detected in both tumor and adjacent non-tumor tissues. We observed that the methylation intensity was higher in tumor tissues compared to adjacent non-tumor tissues (*P*=0.012) (Figure 3B). In addition, we compared the status and intensity of *SOCS3* promoter methylation between tissue samples (tumor and non-tumor) with and without HBV infection. However, no statistical significance was observed.

### *SOCS3* mRNA expression in primary HCCs

**Figure 4:**
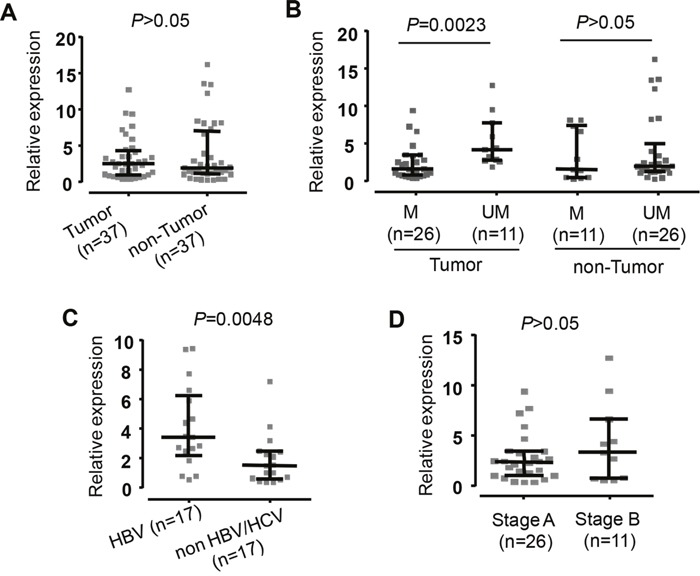
Expression of *SOCS3* mRNA in liver specimens from HCC patients Quantitative real-time PCR (qRT-PCR) analysis presents comparison of SOSC3 mRNA level. **A**. The *SOSC3* mRNA level in the tumor tissues and adjacent non-tumor tissues. **B**. The *SOSC3* mRNA level in the tumor and in non-tumor tissues with methylation (M) and in the tumor and in non-tumore tissues with un-methylation (UM). **C**. The *SOSC3* mRNA level in patients positive for HBV and in patients negative for both HBV and HCV. **D**. The *SOSC3* mRNA level in patients at early HCC stage (Stage A) and in patients at intermediate HCC stage (Stage B). The *GAPDH* gene was used as a reference gene. The data are shown as the medians with inter-quartile range. *P* values were calculated by Mann-Whitney test.

The mean levels of *SOCS3* mRNA expression did not differ significantly between tumor and non-tumor tissues (Figure 4A and Supplementary Figure 3). The hypermethylated tumor tissues had significantly decreased SOCS3 mRNA expression than non hypermethylated tumor tissues (P=0.0023). However, there were no significant differences in SOCS3 mRNA expression between hypermethylated tumor and non-tumor liver tissues. (Figure 4B and Supplementary Figure 3). Our data demonstrate that the hypermethylation status in the *SOCS3* promoter is associated with downregulation of the *SOCS3* mRNA expression in tumor tissues.

SOCS3 mRNA expression discriminating between HCC and non-HCC tissue samples showed that *SOCS3* mRNA expression was significantly higher in HBV-related HCC tissues compared to non-HBV-related HCC tissues (*P*=0.0048) (Figure 4C and Supplementary Figure 3). In order to examine whether *SOCS3* mRNA expression was associated with the development of liver cancer, we analyzed *SOCS3* mRNA expression according to the BCLC staging classification. However, *SOCS3* mRNA expression was not different between stage A and B HCC tissues (Figure 4D and Supplementary Figure 3).

## DISCUSSION

The negative regulator SOCS3 is a key player in the modulation of the JAK/STAT signaling that control a number of inflammatory cytokines such as IL6 and IL16 [6] and is involved in infectious diseases and cancers [13, 14]. In addition, gene silencing mediated by aberrant methylation of CpG islands in the *SOCS3* promoter frequently occurs in malignancies [16, 21]. In this study, we investigated the possible association of *SOCS3* promoter variants with the progression of HBV-related liver diseases and *SOCS3* methylation with HBV-induced HCC. We showed that the *SOCS3* promoter variants are associated with HBV infection and HBV-related liver diseases. *SOCS3* mRNA expression was higher in tumor tissues infected with HBV than non-infected tumor tissues. The aberrant methylation of the CpG islands in the *SOCS3* promoter is associated with relatively low mRNA expression in tumor tissues.

This first study reports on the association of *SOCS3* variants with HBV susceptibility and progression of HBV-related liver diseases. We have shown that the variants rs111033850T/C and rs12953258C/A are associated with HBV infection and the progression of HBV-related liver diseases. Particularly, the variant rs111033850T/C shows a heterozygous advantage in HBV susceptibility but might be a risk factor for the disease progression. The contribution of the minor allele *rs111033850C* to the increased risk of HCC in CHB patients is through the gene dose manner. In studies on hepatitis C, the *rs4969170AA* genotype was associated with antiviral IFN-α resistance with increased *SOCS3* expression in HCV patients [14, 18]. The rs4969170A/G polymorphism was associated with HCV treatment-induced neutropenia and thrombocytopenia in antiviral therapy with pegylated interferon alpha [17]. The rs4969170A/G polymorphism was associated with clinical features and prognosis of HCC after surgical treatment [22]. Our results support earlier findings that *SOCS3* polymorphisms influence liver disease progression by modulating the SOCS3 protein expression and thus down-regulate the JAK/STAT signaling.

In this study, we showed that HBV-DNA loads were associated with the *SOCS3* promoter polymorphisms rs111033850T/C and rs12953258C/A. The HBV-DNA loads are an important and independent risk factor for liver disease progression in CHB patients [23, 24]. The effects of HBV replication during HBV persistence were regulated by many host factors [25]. Previous studies have shown that the control of HBV replication was regulated by JAK/STAT signaling, which is activated by IFNs [15, 26, 27]. IFNs play a central role in control of viral replication including HBV [28] and IFNs are controlled by JAK/STAT signaling, which is regulated by SOCS3 protein [9]. Therefore, *SOCS3* promoter variants might have contributed to control of HBV replication through IFN signaling, which is also regulated by JAK/STAT signaling. However, the effects of *SOCS3* and its variants on the cytokine signaling that subsequently influence the HBV replication are required further studies.

Cytokines are involved in cell communication and are required for the defense against hepatitis viruses [28]. Previous study has shown that the HCV core protein impairs IFN-α-induced signal transduction via induction of *SOCS3* expression [29] and therefore influences the outcome of antiviral therapy [30]. SOCS3 was overexpressed in liver tissues and was strongly associated with severity of hepatic inflammation in CHB patients [15, 27]. In accordance, SOCS3 overexpression was observed in liver tissues from HBV-infected patients rather than non-HBV patients. These results may indicate that HBV can induce *SOCS3* expression, which in turn inhibits IFN signaling transduction resulting in the progression of liver diseases and failure of IFN treatment of HBV infection [27].

HCC development is often due to chronic liver injury, inflammation, and cirrhosis caused by the persistence of HBV infection. However, the interaction between HBV and SOCS3 in infected hepatocytes has not been clearly understood. DNA hypermethylation in the promoter region can lead to the silence of *SOCS3* in HCC [16, 31]. *SOCS3* silencing by promoter methylation is possibly involved in the progression of HBV-related liver cancer. In line with previous studies, our results showed that aberrant methylation in the *SOCS3* promoter region was observed more frequently in tumor tissues compared to adjacent non-tumor tissues. Hypermethylation status in the *SOCS3* promoter region may be a crucial factor for HCC development. However, *SOCS3* expression in tumor and adjacent non-tumor tissues was not significantly different, suggesting that other factors such as phosphorylation, acetylation and microRNAs may also involve in the regulation of *SOCS3* expression during progression of liver diseases [32]. In addition, both *SOCS3* methylation status and intensity were not significantly different between tissue samples (tumor and non-tumor) from patients with and without HBV infection. This indicates that HBV may not promote DNA methylation of host genes in infected hepatocytes. However, further studies are needed to verify this preliminary observation since the number of samples used for this analysis was rather small.

Although our data indicate that SOCS3 expression is associated with HBV infection and may involve in the progression of HBV-related liver diseases, the study has several limitations. A limited number of HCC tumor and non-tumor tissues were utilized. Due to the study design as a case-control study, *SOCS3* expression over the course of HBV infection were not assessed longitudinally and therefore the causative effect of *SOCS3* expression on progression of HBV-related liver diseases could not conclusively be determined. The insufficiency of some clinical and laboratory parameters such as HBV serology tests and HBV genotypes may weaken the findings indicating the crucial role of SOCS3 in the immune response to HBV infection and the disease outcomes.

In conclusion, the *SOCS3* promoter variants rs111033850 and rs12953258 are associated with HBV infection and HBV-related liver diseases. DNA methylation in the *SOCS3* promoter region is related to the regulation of *SOCS3* expression and occurs frequently in HCC tumors of HBV-infected patients. Our study suggests that *SOCS3* polymorphisms and methylation play an important role in regulation of *SOCS3* expression and thus influences the progression of HBV-related liver diseases.

## MATERIALS AND METHODS

### Patients and liver specimens

878 unrelated Vietnamese HBV-infected patients were randomly recruited in a case-control design at 108 Military Central Hospital and 103 Military Hospital of the Vietnam Military Medical University, Hanoi, Vietnam, between 2012 and 2013. With this sample size, we can detect the significance of common studied *SOCS3* variants according to the sample size estimation based on the 95% confidence interval, the lowest detection rate of minor allele set at the lowest value of 5%, a significance level of 5% and a power >90%. Patients were assigned to subgroups of disease based on clinical manifestations and liver function tests. Subgroups included chronic hepatitis (CHB, n=212), liver cirrhosis (LC, n=243), hepatocellular carcinoma (HCC, n=220) and patients with LC and HCC (LC+HCC, n=203). The diagnostic criteria for the CHB patients and the HBV-related LC were previously described [33]. The HBV-related HCC group was characterized as patients infected with HBV and was diagnosed based on the American Association for the Study of Liver Diseases (AASLD) practice guideline for HCC [34]. The patients with LC and HCC were characterized if the patients showed clinical manifestations and laboratory tests of both LC and HCC. The patients with LC were also categorized as Child-A, Child-B and Child-C based on Child-Pugh scores [35]. None of these HBV-infected patients had a history of alcohol or drug abuse. All participants were confirmed negative for anti-HCV and anti-HIV by ELISA assays. HBV-DNA loads and liver function tests including alanine transaminase (ALT), aspartate transaminase (AST), total bilirubin and direct bilirubin, albumin, prothrombin were quantified. 272 blood samples from healthy individuals were collected from blood bank as the control group. In addition, we analyzed 37 dyads of liver specimens (tumor and adjacent non-tumor) collected from HCC patients who underwent surgery at the 108 Military Central Hospital between 2013 and 2014. The HCC patients who underwent surgery were independent from 878 HBV patients. HCC was confirmed by histology and classified based on the BCLC classification [36]. All specimens were frozen at -80°C until use.

### Ethics statement

Informed written consent was obtained after explanation of the study at the time of sampling from all participants. The study was approved by the institutional review board of the 108 Military Central Hospital and the 103 Military Hospital of the Vietnam Military Medical University, Hanoi, Vietnam.

### Genotyping of *SOCS3* promoter variants

Genomic DNA was isolated from blood using DNA purification kits (Qiagen, Hilden, Germany). The *SOCS3* promoter region (nucleotides -1109 to -772) including two pre-described SNPs (rs111033850 and rs12953258) were amplified by PCR using primer pair *SOCS3_PrF* and *SOCS3_PrR* (Supplementary Table 1). PCR components, thermal conditions and sequencing procedures are presented in the Supplementary Materials.

### *SOCS3* promoter methylation analysis

Liver tissues were grounded using liquid nitrogen. Genomic DNA was extracted from liver powder using DNA purification kit (Qiagen, Hilden, Germany). Extracted DNA (2μg) was subjected to bisulfite conversion using EZ DNA Methylation-Direct™ Kit (Zymo Research Corp, the USA), according to the manufacturer’s protocol. Three different fragments were amplified from bisulfite-treated genomic DNA using three specific primer sets (Figure 2 and Supplementary Table 1). The PCR products were subsequently cloned into TOPO-TA pCR2.1 vector (Life Technologies, the USA). Eight clones were randomly picked from each transformation (each tissue sample) and were analyzed for methylation by direct sequencing.

### *SOCS3* mRNA expression

Total RNA was extracted from 37 dyads of liver biopsy tissues with Trizol reagent (Life Technologies, the USA). RNA was reverse transcribed into cDNA using QuantiTect Reverse Transcription Kit (Qiagen GmbH, Hilden, Germany). cDNA quantification was performed by qRT-PCR and *GAPDH* (glyceraldehyde-3-phosphate dehydrogenase) gene was used as a reference gene. The specific primers used for evaluating *SOCS3* mRNA expression as well as the PCR components and thermal conditions of qRT-PCR are presented in the Supplementary Materials. Calculation of normalized gene expression was based upon the ΔΔCT method.

### Statistical and genetic analysis

All statistical analysis was performed using R version 3.1.2 (http://www.r-project.org) and GraphPad Prism 6 (http://www.graphpad.com). Genotype and allelic frequencies were determined by simple gene counting and the haplotype frequency was estimated using the expectation-maximum algorithm method implemented in the Arlequin v.3.5.2.2. The deviations from Hardy-Weinberg equilibrium were calculated for each group. We used a binary logistic regression adjusted for age and gender to analyze association of *SOCS3* variants with HBV-related liver diseases applying for different genetic models. In the binary logistic regression model, the disease subgroups and control group are considered as dependent variables. The genetic data are considered as independent variables (predictors) while age (treated as a continuous variable) and gender (treated as a binary variable) are independent confounding factors. In addition, the Cochran-Armitage test for trend was used to examine the gene dose effect of risk allele. Fisher’s exact test was used to test the difference of categorical variables between two groups. Student's t-test and Mann Whitney Wilcoxon test were used to compare the parametric and non-parametric data of quantitative variables between two groups, respectively. Kruskal-Wallis test was used to compare non-parametric data of quantitative variables among more than two groups. The level of significance was set at a value of *P*< 0.05.

## SUPPLEMENTARY MATERIALS FIGURES AND TABLES



## Abbreviations

CHB: chronic hepatitis B
HBV: hepatitis B virus
HC: healthy control
HCC: hepatocellular carcinoma
LC: liver cirrhosis
SNP: single nucleotide polymorphism
SOCS3: suppressor of cytokine signaling-3.
