# Supplementary material for: SOCS3 genetic variants and promoter hypermethylation in patients with chronic hepatitis B
Source: Oncotarget. 2017 Feb 4;8(10):17127–39. doi: 10.18632/oncotarget.15083 (PMC5370028; doi:10.18632/oncotarget.15083)
Supplement: Supplementary file 1 [file oncotarget-08-17127-s001.pdf]

SOCS3 genetic variants and promoter hypermethylation in patients with chronic hepatitis B

SUPPLEMENTARY DATA

SUPPLEMENTARY MATERIALS AND METHODS

Genotyping of *SOCS3* promoter variants

Genomic DNA was isolated from whole blood using a DNA purification kit (Qiagen, Hilden, Germany). The *SOCS3* promoter region (nucleotides -1109 to -772) including two pre-described SNPs (rs111033850 and rs12953258) were amplified by PCR using primer pair *SOCS3_PrF* and *SOCS3_PrR* (Supplementary Table 1). PCR amplifications were carried out in a 25µl reaction volume containing (Qiagen): 1X PCR buffer, 0.2mM dNTPs, 1mM MgCl2, 0.15mM of each primer, 1unit of Taq polymerase and 50ng of genomic DNA. The cycling conditions were as follows: denaturation at 95°C for 5min, followed by 40 cycles of three-step cycling with denaturation at 94°C for 30s, annealing at 63°C for 35s, and extension at 72°C for 45s and a final extension at 72°C for 7min. PCR products were purified using Exo-SAP-IT (USB, Affymetrix, USA). 5µl of purified PCR products were used as templates. The sequencing was performed using the BigDye terminator v.1.1 cycle sequencing kit (Applied Biosystems, Foster city, USA) on an ABI 3130XL DNA sequencer according to the manufacturer's instructions. Sequencing results were finally analysed using vector NTI v.10 software.

SOCS3 mRNA expression

Total RNA was extracted from 37 dyads of liver biopsy tissues with Trizol reagent (Life Technologies). RNA was reverse transcribed into cDNA using QuantiTect Reverse Transcription Kit (Qiagen GmbH, Hilden, Germany). Quantification of cDNA was performed by qRT-PCR using SYBR Green PCR mix (Bioline, Germany). All reactions were performed in triplicate using the LightCycler®480 real-time PCR system (Roche, Switzerland). The *GAPDH* (glyceraldehyde-3-phosphate dehydrogenase) gene was used as a reference gene. In addition, the *TGFBRAP-1* gene (transforming growth factor, beta receptor associated protein 1) was selected as an additional reference gene using Genevestigator (https://genevestigator.com/gv/). The specific primers used for evaluating the *SOCS3* mRNA expression were presented in the Supplementary Table 1. The thermal cycling conditions were as follows: 2min at 95°C followed by 45 cycles of denaturation at 95°C for 5s and annealing at 58°C for 10s and extension at 72°C for 20s. The specificity of each reaction was confirmed by melting curve analysis. Calculation of normalized gene expression was based upon the ΔΔCT method. The fold change in *SOCS3* expression was normalized to the expressed reference genes and then compared to the mean level expression in non-tumor tissues as the calibrator sample as follows: $2^{-\Delta\Delta CT}$, where $\Delta\Delta Ct=(Ct_{socs3}-Ct_{ref})_{cancer\text{-}sample}-(Ct_{socs3}-Ct_{ref})_{calibrator\text{-}sample}$ [1].

REFERENCES

1. Livak KJ, Schmittgen TD. Analysis of relative gene expression data using real-time quantitative PCR and the 2(-Delta Delta C(T)) Method. Methods. 2001; 25:402-8.
2. He B, You L, Uematsu K, Zang K, Xu Z, Lee AY, Costello JF, McCormick F, Jablons DM. SOCS-3 is frequently silenced by hypermethylation and suppresses cell growth in human lung cancer. Proc Natl Acad Sci U S A. 2003; 100:14133-8.
3. Niwa Y, Kanda H, Shikauchi Y, Saiura A, Matsubara K, Kitagawa T, Yamamoto J, Kubo T, Yoshikawa H. Methylation silencing of SOCS-3 promotes cell growth and migration by enhancing JAK/STAT and FAK signalings in human hepatocellular carcinoma. Oncogene. 2005; 24:6406-17.
4. Walsh MJ, Jonsson JR, Richardson MM, Lipka GM, Purdie DM, Clouston AD, Powell EE. Non-response to antiviral therapy is associated with obesity and increased hepatic expression of suppressor of cytokine signalling 3 (SOCS-3) in patients with chronic hepatitis C, viral genotype 1. Gut. 2006; 55:529-35.
5. Imai K, Kamio N, Cueno ME, Saito Y, Inoue H, Saito I, Ochiai K. Role of the histone H3 lysine 9 methyltransferase Suv39 h1 in maintaining Epsteinn-Barr virus latency in B95-8 cells. FEBS J. 2014; 281:2148-58.

SUPPLEMENTARY FIGURES AND TABLES

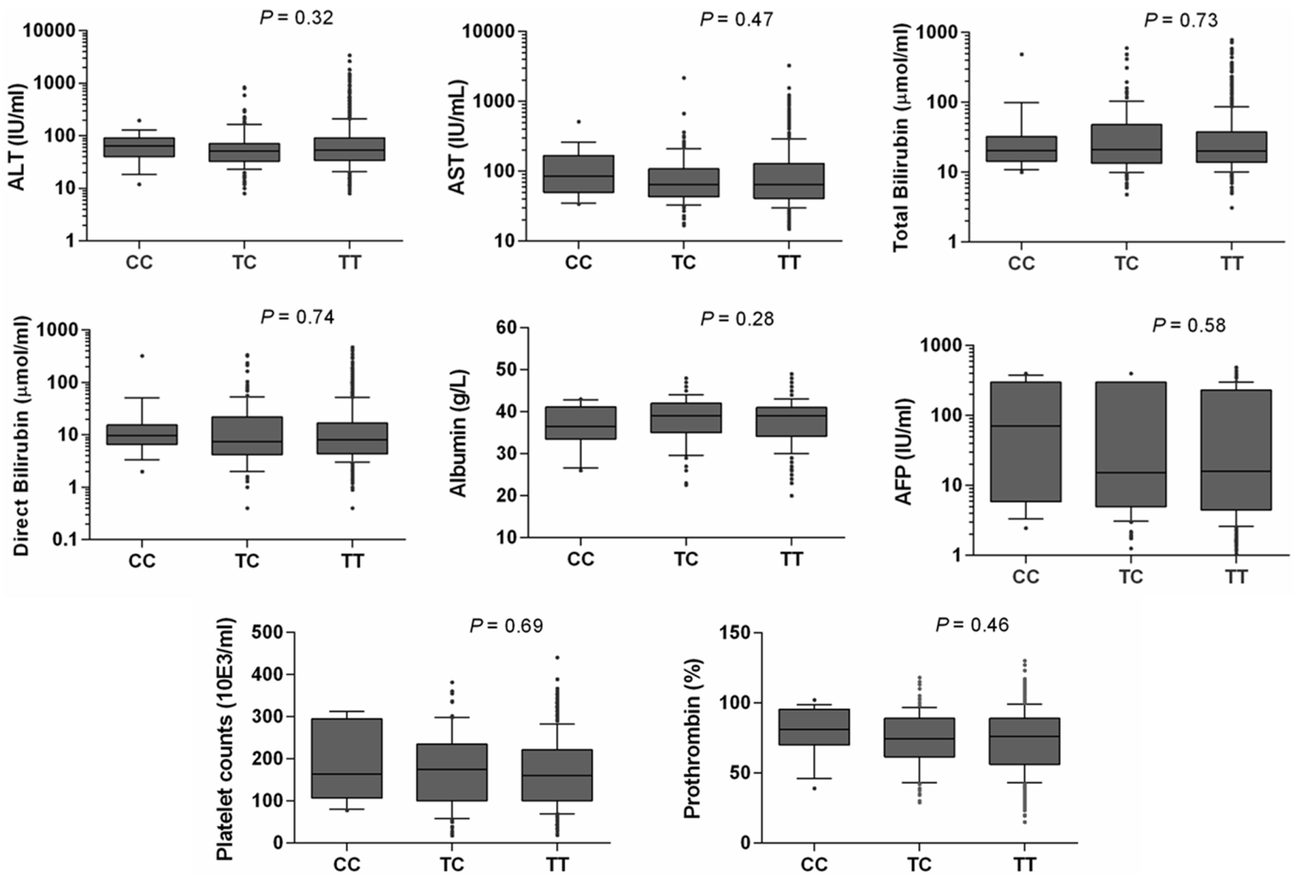

Supplementary Figure 1: Association of clinical parameters with *SOCS3* rs111033850T/C variants. Box-plots illustrate medians with 25 and 75 percentiles with whiskers to 10 and 90 percentiles; P values were calculated by Krusskal - Wallis test.

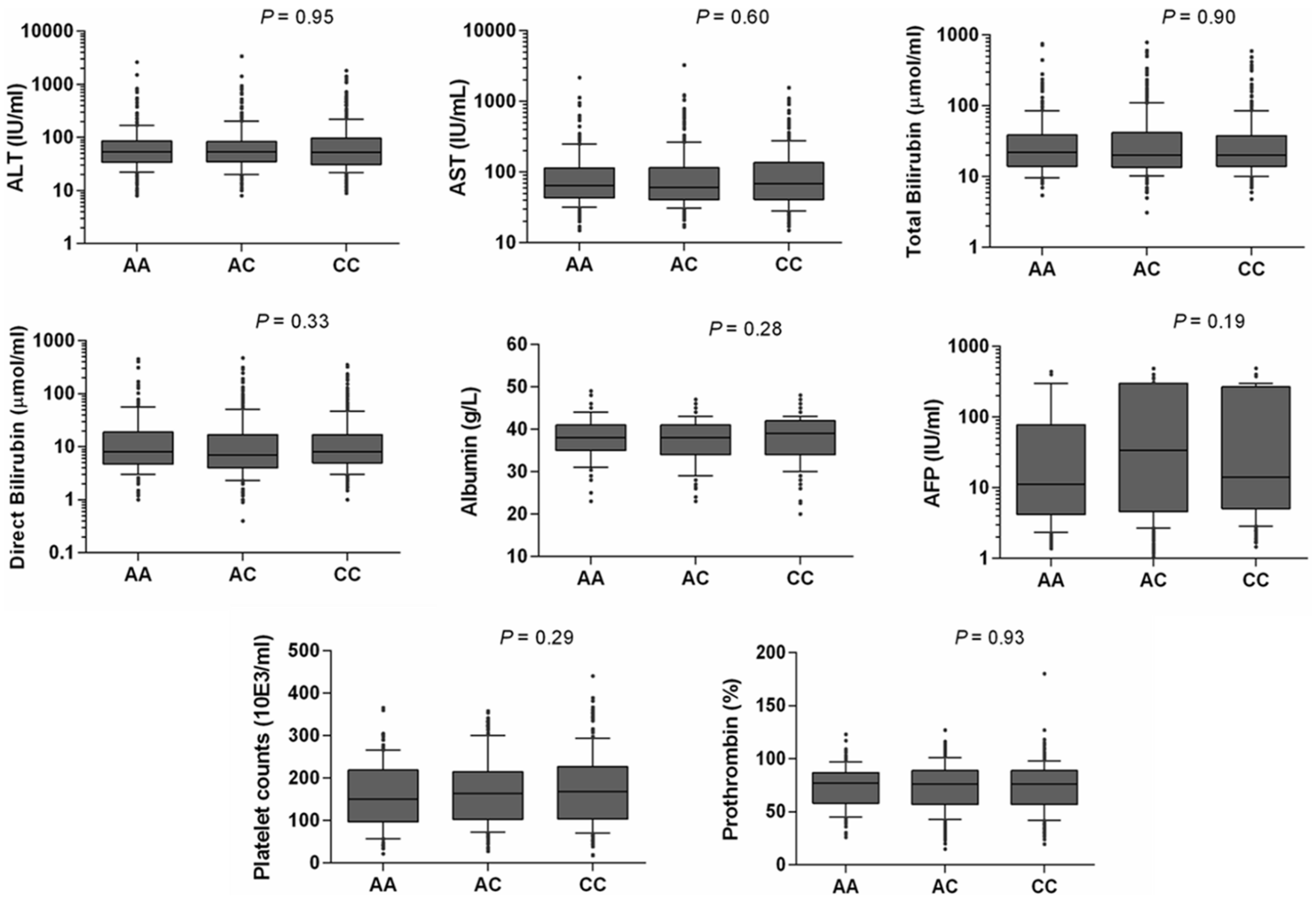

Supplementary Figure 2: Association of clinical parameters with *SOCS3* rs12953258C/A variants. Box-plots illustrate medians with 25 and 75 percentiles with whiskers to 10 and 90 percentiles; P values were calculated by Krusskal - Wallis test.

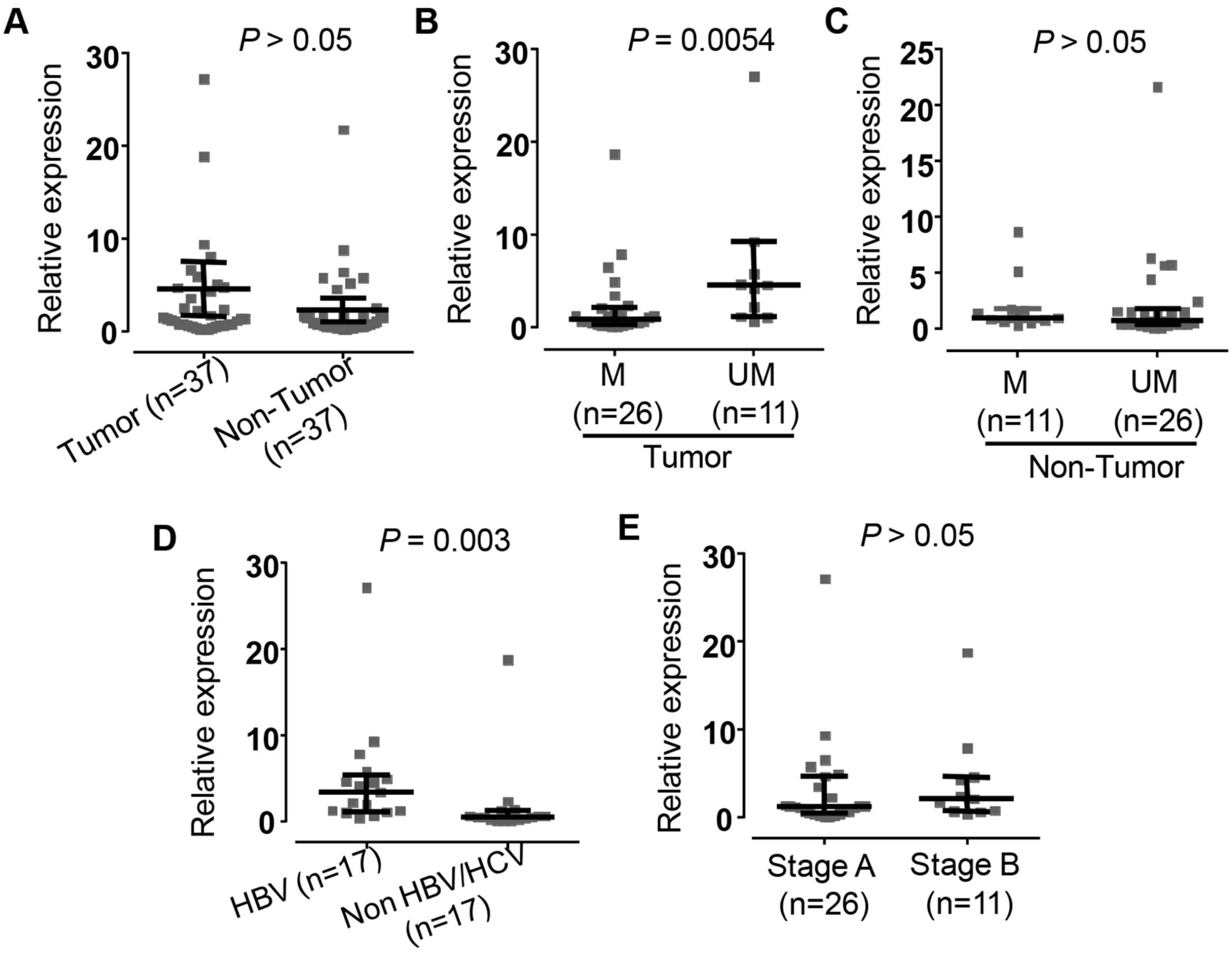

Supplementary Figure 3: Expression of *SOCS3* mRNA in liver specimens from HCC patients. Quantitative real-time PCR (qRT-PCR) analysis presents comparison of *SOSC3* mRNA level. **A.** The *SOSC3* mRNA level in the tumor tissues and adjacent non-tumor tissues. **B, C.** The *SOSC3* mRNA level in the tumor and in non-tumor tissues with methylation (M) and in the tumor and in non-tumore tissues with un-methylation (UM). **D.** The *SOSC3* mRNA level in patients positive for HBV and in patients negative for both HBV and HCV. **E.** The *SOSC3* mRNA level in patients at early HCC stage (Stage A) and in patients at intermediate HCC stage (Stage B). The *TGFBRAP-1* gene was used as a reference gene. The data are shown as the medians with inter-quartile range. P values were calculated by Mann-Whitney test.

Supplementary Table 1: Primers used in this study

Primer	Sequence	Application
SOCS3_PrF	5'- CCG CGC TCA GCC TTT CTC TGC TGC GA-3'	*SOCS3* genotyping
SOCS3_PrR	5'-AGT CCA CAA AGG AGC CTT CGC GCG CG-3'	*SOCS3* genotyping
SOCS3_Fr1_F	5'-GTG TAG AGT AGT GAT TAA ATA-3'	*SOCS3* promoter methylation
SOCS3_Fr1_R	5'-TCC TTA AAA CTA AAC CCC CTC-3'	*SOCS3* promoter methylation
SOCS3_Fr2_F	5'-GAT TYG AGG GGG TTT AGT TTT AAG GA-3'	*SOCS3* promoter methylation
SOCS3_Fr2_R	5'-CCA CTA CCC CAA AAA CCC TCT CCT AA-3'	*SOCS3* promoter methylation
SOCS3_Fr3_F	5'- GGG AAG GGG TTG TTY GGG GTT ATT TTG -3'	*SOCS3* promoter methylation
SOCS3_Fr3_R	5'- CAA ACT AAT ATC CAA AAA ACA ACT CAT CC -3'	*SOCS3* promoter methylation
SOCS3_Exp_F	5'- CCC TCG CCA CCT ACT GAA -3'	SOCS3 mRNA expression
SOCS3_Exp_R	5'- TCC GAC AGA GAT GCT GAA GA -3'	SOCS3 mRNA expression
GAPDH_F	5'-TGCACCACCAACTGCTTAGC-3'	SOCS3 mRNA expression
GAPDH_R	5'-GGCATGGACTGTGGTCATGAG-3'	SOCS3 mRNA expression
TGFBRAP-1_F	5'-GCG GCT GTG TCC TTT CCA TA-3'	SOCS3 mRNA expression
TGFBRAP-1_R	5'-GCG TCT GCT TCT GTT GCT GAT-3'	SOCS3 mRNA expression

The specific primer pair *SOCS3_PrF* and *SOCS3_PrR* were designed to amplify nucleotides -1109 to -772 from start codon and were used for amplification and sequencing of the SOCS3 promoter region. Primers for fragment 1 were *SOCS3_Fr1_F* and *SOCS3_Fr1_R* [2], primers for fragment 2 were *SOCS3_Fr2_F* and *SOCS3_Fr2_R* [3], and primers for fragment 3 were *SOCS3_Fr3_F* and *SOCS3_Fr3_R*. These primers were designed to amplify nucleotides -1091 to -679; - 704 to -186, and -295 to +60, respectively. The PCR amplification of the three fragments were performed as described previously (2;3). The specific primers used for evaluating the expression of *SOCS3* mRNA were *SOCS3_Exp_F* and *SOCS3_Exp_R* [4]. Primers used for the reference genes *were GAPDH_F* and *GAPDH_R* (*GAPDH)* [5] and were *TGFBRAP-1_F* and *TGFBRAP-1_R* (*TGFBRAP-1*).

Supplementary Table 2: Allelic and genotypic frequencies of *SOCS3* variants in sub-HBV patient groups and healthy controls

SOCS3 variants	CHB (%)	LC (%)	HCC (%)	HCC+LC (%)	HC (%)	CHB vs. HC		LC vs. HC		HCC vs. HC		HCC+LC vs. HC	
	n=212	n=243	n=220	n=203	n=272	OR (95%CI	*P* value	OR (95%CI	*P* value	OR (95%CI	*P* value	OR (95%CI	*P* value
**rs111033850T/C**													
**Genotype**													
TT	190(89.6)	198(81.5)	178(80.9)	162(79.8)	191(70.3)	Reference		Reference		Reference		Reference	
TC	16(7.5)	43(17.7)	36(16.4)	35(17.2)	76(27.9)	**0.2(0.1-0.4)**	**< .0001**	**0.6 (0.4-0.9)**	**0.02**	**0.6(0.4-1.0)**	**0.045**	**0.6(0.3-0.9)**	**0.03**
CC	6(2.9)	2(0.8)	6(2.7)	6(3.0)	5(1.8)	1.2(0.4 - 4.1)	0.73	1.7(0.3 - 9.9)	0.5	1.26 (0.3-5.1)	0.7	1.9(0.4 -9.1)	0.43
**Allele**													
T	396(93.4)	439(90.3)	392(89)	359(88.4)	458(84.2)	Reference		Reference		Reference		Reference	
C	28(6.6)	47(9.7)	48(11)	47(11.6)	86(15.8)	**0.4(0.2-0.6)**	**< .0001**	**0.6(0.4-0.9)**	**0.03**	**0.49(0.3-0.8)**	**0.001**	**0.7(0.5-1.2)**	0.21
**Dominant**													
TT	190(89.6)	198(81.5)	178(80.9)	162(79.8)	191(70.3)	Reference		Reference		Reference		Reference	
TC & CC	22(10.4)	45(18.5)	42(19.1)	41(20.2)	81(29.7)	**0.3(0.2-0.5)**	**< .0001**	**0.6(0.4-0.9)**	**0.02**	0.6(0.4-1.1)	0.07	0.6(0.4-1.1)	0.08
**Recessive**													
TT & TC	206(97.2)	241(99.2)	214(97.3)	197(79.8)	267(98.2)	Reference		Reference		Reference		Reference	
CC	6(2.9)	2(0.8)	6(2.7)	6(3.0)	5(1.8)	1.6 (0.5-5.3)	0.45	0.7(0.1 - 4.0)	0.7	1.4(0.4-5.6)	0.6	2.1(0.4-10.2)	0.34
**rs111033850C/A**													
**Genotype**													
CC	94(44.3)	88(36.2)	86(39.1)	75(36.9)	101(37.1)	Reference		Reference		Reference		Reference	
AC	72(34)	88(36.2)	88(40)	84(41.4)	140(51.5)	**0.6(0.4 - 0.8)**	**0.00014**	0.8(0.5 - 1.2)	0.21	0.8(0.5-1.2)	0.27	0.9(0.5-1.5)	0.61
AA	46(21.7)	67(27.6)	46(20.9)	44(21.7)	31(11.4)	1.63(1 - 2.8)	0.08	**3.0(1.69-5.32)**	**< 0.0001**	**1.8(1.0-3.3)**	**0.047**	**2.8 (1.3-5.6)**	**0.004**
**Allele**													
C	260(61.3)	264 (54.3)	260 (59.1)	172(42.4)	342(62.9)	Reference		Reference		Reference		Reference	
A	164(38.7)	222 (45.7)	180 (40.9)	234(57.6)	202 (37.1)	1.1(0.8-1.4)	0.58	**1.6 (1.2-2.1)**	**0.001**	1.2(0. 9 - 1.6)	0.25	**1.5(1.1-2.1)**	**0.022**
**Dominant**													
CC	94(44.3)	88 (36.2)	86(39.1)	75(36.9)	101(37.1)	Reference		Reference		Reference		Reference	
AC & AA	118(55.7)	155 (63.8)	134(60.9)	128(63.1)	171(62.9)	0.7(0.5-1.1)	0.12	1.2(0.8-1.7)	0.48	1(0.6 - 1.5)	0.86	1.2(0.7-1.9)	0.48
**Recessive**													
CC & AC	166(78.3)	176(72.4)	174(79.1)	159(78.3)	241(88.6)	Reference		Reference		Reference		Reference	
AA	46(21.7)	67(27.6)	46(20.9)	44(21.7)	31(11.4)	**2.2(1.3-3.6)**	**0.0019**	**3.5(2.1-5.7)**	**< 0.0001**	**2.0(1.1-3.6)**	**0.015**	**3(1.6-5.7)**	**0.0007**

CHB: Chronic hepatitis B; LC: Liver cirrhosis; HCC: Hepatocellular carcinoma; HC: Healthy control; Cases = all HBV infected patients; n= Number of chromosomes; OR: adjusted Odd Ratio; ORs and *P* values were calculated by using binary logistic regression model adjusted for age and gender. Bold values present the statistical significance.
